# Assessment of left ventricular systolic and diastolic function in patients with sarcoidosis with and without cardiac involvement by magnetic resonance feature tracking at 1.5T: a follow up study

**DOI:** 10.1186/1532-429X-18-S1-O102

**Published:** 2016-01-27

**Authors:** Darius Dabir, David Meyer, Rami Homsi, Julian A Luetkens, Christian Marx, Folke Kluenker, Daniel Kuetting, Hans H Schild, Daniel K Thomas

**Affiliations:** Radiology, University of Bonn, Bonn, Germany

## Background

Cardiac involvement in patients with sarcoidosis is an independent predictor of mortality and associated with very poor prognosis. Inflammatory processes within the myocardium as well as subsequent fibrotic myocardial alterations may lead to diastolic dysfunction and at worst sudden death resulting from ventricular tachy-arrhythmias or conduction block. Diastolic dysfunction as a sign of left ventricular (LV) impairment can be assessed accurately by the measurement of early diastolic strain rate (EDSR). In this follow up study we performed cardiovascular magnetic resonance (CMR) feature tracking (FT) in patients with and without cardiac involvement of sarcoidosis in order to investigate its impact on systolic and especially diastolic function over time.

## Methods

Patients with systemic sarcoidosis were consecultively enrolled into the study. They underwent clinically routine CMR exams on a 1.5 Tesla Philips INGENIA scanner. CMR exams were classified as "cardiac involvement" if at least one of the following pathologies occured: pathological relative enhancement, late enhancement findings consistent with (post-) inflammatory changes, myocardial edema, pericardial effusion. Patients with cardiac involvement underwent a follow up CMR exam within a time frame of 36 ± 3,5 months. According to CMR results patients were divided into 4 different groups (Gr.): group 1 (n = 14): patients with positive findings in both exams, group 2 (n = 9): patients with positive findings only in the initial exam; group 3 (n = 37): patients with sarcoidosis but inconspicuous initial CMR exam and group 4 (n = 20): a control group of healthy age matched volunteers with no medical history of sarcoidosis or cardiac disease. In addition in each CMR exam FT derived systolic and diastolic circumferential strain parameters were calculated from SSFP-cine images in midventricular short axis, which were acquired prior to contrast agent injection. Peak systolic strain rate (PSSR), peak diastolic strain rate (PDSR), Peak systolic circumferential strain (PSECC) as well as early diastolic strain rate (EDSR) were calculated and compared between groups. Patient characteristics are demonstrated in table 1.

**Table 1:** Table 1

	Initial exam							
	PSSR	PDSR	PSECC	EDSR	Age (y)	LVEF (%)	LVEDV (ml)	IVSD (mm)
Group1 (n= 14)	-1,09 ± 0,75	1,14 ± 0,35	-18,43 ± 4,27	53,24 ± 15,26	50,57 ± 13,51	62,15 ± 8,87	120 ± 24,42	10,63 ± 1,66
Group2 (n = 9)	-1,49 ± 0,37	1,42 ± 0,42	-22,21 ± 6,47	75,1 ± 22,62	51,89 ± 5,88	68,33 ± 7,75	106,33 ± 6	10,69 ± 1,69
Group3 (n = 37)	-1,51 ± 0,39	1,36 ± 0,1	-24,33 ± 3,5	77,25 ± 5,06	50,78 ± 12,66	64,84 ± 4,76	116,53 ± 31,64	9,47 ± 1,62
Group4 (n = 20)	-1,51 ± 0,28	1,37 ± 0,48	-24,53 ± 4,38	79,74 ± 25,59	59,32 ± 28,99	61,88 ± 7,07	135,99 ± 9,19	9,7 ± 1,84
		Significance (p-value)						
Group1 vs. Group2	0,1813	0,1153	0,1132	0,0133				
Group1 vs. Group3	0,0105	0,1440	0,0010	0,0006				
Group1 vs. Group4	0,0248	0,1347	0,0002	0,0014				

FT results of group 1-4 including 2-tailed t-test results and patient characteristics.

## Results

EDSR in group1 patients was significantly lower (53,24 ± 15,26) compared to all other groups (Gr.2: 75,1 ± 22,62, Gr.3: 77,25 ± 5,06, Gr.4: 79,74 ± 25,59 respecitvely; p < 0,05; table 1). Furthermore PSSR and PSECC were significantly lower in group 1 in comparison to group 3 and 4 (p < 0,05; table 2). In both group 1 and 2 there was no significant difference between initial CMR exam and follow-up scan regarding PSSR, PDSR, PSECC or EDSR (p > 0,05; table 3).

**Table 2:** Table 2

	Initial exam	Follow up	Initial exam	Follow up	Initial exam	Follow up	Initial exam	Follow up	Significance (p-value)
	PSSR		PDSR		PSECC		EDSR		
Group1 (n= 14)	-1,09 ± 0,75	-1,4 ± 0,42	1,14 ± 0,35	1,24 ± 0,39	-18,43 ± 4,27	-19,25 ± 13,94	53,24 ± 15,26	64,84 ± 20,39	p > 0,05
Group2 (n = 9)	-1,49 ± 0,37	-1,4 ± 0,42	1,42 ± 0,42	1,2 ± 0,39	-22,21 ± 6,47	-19,25 ± 13,94	75,1 ± 22,62	75,62 ± 22,62	p > 0,05

FT results of group 1 and group 2 regarding initial exam and follow-up including 2-tailed t-test results.

## Conclusions

This study reveals 2 major findings. First patients with consistent cardiac involvement of sarcoidosis have a significantly reduced EDSR. Second diastolic function in patients with sarcoidosis and significantly impaired EDSR is not likely to recover over time.

